# Development of a three-dimensional evaluation system for outpatient healthcare quality in stomatological hospitals

**DOI:** 10.3389/fpubh.2026.1773077

**Published:** 2026-04-15

**Authors:** Wei Liu, Haiqian Chen, Qingbin Zhang

**Affiliations:** 1 Department of Medical, School and Hospital of Stomatology, Guangdong Engineering Research Center of Oral Restoration and Reconstruction, Guangzhou Key Laboratory of Basic and Applied Research of Oral Regenerative Medicine, Guangzhou Medical University, Guangzhou, China; 2 School and Hospital of Stomatology, Guangdong Engineering Research Center of Oral Restoration and Reconstruction, Guangzhou Key Laboratory of Basic and Applied Research of Oral Regenerative Medicine, Guangzhou Medical University, Guangzhou, China

**Keywords:** Delphi method, medical quality assessment system, outpatient medical quality, stomatological hospital, three-dimensional quality evaluation method

## Abstract

**Objective:**

To develop a three-dimensional (structure-process-outcome) evaluation index system for outpatient healthcare quality tailored to the “large outpatient, small inpatient” operational characteristics of stomatological hospitals in China, addressing the current monitoring gap of over-reliance on outcome measures while neglecting structural and process dimensions.

**Methods:**

A mixed-methods design was employed. Initial dimensions were established through systematic literature review and focus group discussions (*n* = 8). A two-round Delphi expert consultation was conducted with 28 specialists (authority coefficient Cr = 0.824; response rates: 87.5% → 100%) to select indicators using threshold criteria (importance score ≥4.0, coefficient of variation ≤0.25). The Analytic Hierarchy Process was used to calculate indicator weights, and Cronbach’s *α* coefficient was applied to assess reliability.

**Results:**

The final index system comprised 3 first-level indicators, 16 s-level indicators, and 77 third-level indicators. First-level indicator weights were: process quality (0.340) > outcome quality (0.333) > structural quality (0.328), with a consistency ratio CR = 0.041. The highest-weighted third-level indicator was outpatient medical record writing qualification rate (0.044). Expert coordination coefficients improved from 0.209 (first round) to 0.365 (second round) (*p* < 0.001). Cronbach’s *α* coefficients for all dimensions ranged from 0.604 to 0.975.

**Conclusion:**

By shifting quality control checkpoints forward to the care delivery process (highest weight assigned to process quality), this index system facilitates a paradigm shift from “post-hoc remediation” to “proactive prevention” in quality management. It provides an evidence-based tool for standardized quality evaluation, cross-regional benchmarking, and quality monitoring under DRG/DIP payment reforms in stomatological hospitals, offering significant public health administration value.

## 1 Introduction

Oral diseases represent a major public health challenge worldwide and are closely associated with overall health and quality of life. Increasing evidence suggests a bidirectional relationship between periodontal diseases and systemic conditions such as diabetes, cardiovascular disease, and adverse pregnancy outcomes (1–3). Improving oral healthcare quality therefore has important implications for population health. In China, the Fourth National Oral Health Epidemiological Survey revealed that the prevalence of dental caries among Chinese populations aged 5, 12, 35–44, and 65–74 has increased by 5.8, 7.9, 8.0, and 8.3%, respectively, compared to a decade ago. Notably, only 9.1% of middle-aged and older adult(s) individuals possess good periodontal health, indicating the burden of oral diseases has increased substantially. Meanwhile, the utilization rate of oral health services among residents rose by 15.5 to 23.8% over the past decade, reflecting demand for dental services continues to grow with increasing public awareness of oral health (4).

However, the supply of dental healthcare resources remains insufficient and unevenly distributed. The latest national healthcare services survey shows that China has only one licensed dentist for every 7,768 people, just 64.4% of the WHO’s recommended standard of one dentist per 5,000 people. Furthermore, high-quality resources are highly concentrated in specialized dental hospitals (5, 6). Stomatological hospitals serve as the main providers of specialized dental services in China and operate under a “large outpatient–small inpatient” model. More than 90% of clinical services are delivered chairside, meaning that outpatient service quality plays a central role in overall healthcare quality. Despite this importance, in China, current the quality monitoring of dental specialty outpatient services primarily relies on terminal indicators, such as cure rates and complication rates. There is a notable lack of dynamic evaluation tools for element quality (including resource allocation and personnel competency) and process quality (such as diagnostic and treatment procedures and technical standards). This results in fragmented and delayed quality improvement efforts (7). The “2018 National Medical Service and Quality Safety Report” explicitly states that oral medicine, as a primary discipline, lacks a comprehensive ‘structure-process-outcome’ quality evaluation indicator system, making it difficult to achieve refined management and continuous quality improvement (8).

Moreover, current outpatient healthcare quality monitoring in stomatological hospitals in China faces three critical limitations that demand urgent attention. First, unidimensional evaluation scope: existing monitoring systems predominantly focus on outcome quality indicators (e.g., root canal treatment adequacy rate, implant failure rate) while lacking systematic assessment of structural quality (personnel configuration, equipment integrity, institutional completeness) and process quality (standardization of clinical procedures, effectiveness of physician-patient communication, coordination between medical and nursing staff). This imbalance constrains quality improvement to “post-hoc remediation” rather than “proactive prevention” (9). Second, insufficient specialty specificity: generic hospital quality frameworks (e.g., traditional SPO models applied in general hospitals) fail to accommodate the distinctive operational characteristics of stomatological outpatient services—characterized by “large outpatient volume, small inpatient capacity, and chairside-centered operations” (10). Consequently, current indicator systems omit refined evaluation of specialty-specific technical procedures such as four-handed dentistry execution rate, grade-A dental film rate, and oral mucosal disinfection protocols. Third, indicator fragmentation and homogeneity deficits: prior research has predominantly concentrated on single subspecialties or isolated quality dimensions (e.g., orthodontic quality alone or infection control alone), resulting in the absence of unified, cross-institutionally comparable standards covering the complete outpatient workflow (11). This fragmentation impedes horizontal benchmarking and continuous improvement across different stomatological hospitals.

Internationally, the Dental Quality Alliance (DQA) under the American Dental Association (ADA) has developed dental quality indicator systems; however, these rely primarily on administrative claims data, emphasizing accessibility metrics (e.g., annual dental examination rates among diabetic patients) and basic process indicators within a payment and accountability framework (12). This approach lacks direct evaluation of on-site management elements (institutional responsibility systems, personnel emergency response capabilities) and specialty-specific technical procedures. Meanwhile, the World Health Organization’s oral health evaluation framework focuses on population-level epidemiological indicators (e.g., DMFT index, caries-free rate), designed for public health surveillance rather than internal quality management within healthcare institutions (13). Therefore, constructing a novel evaluation index system that aligns with international quality management theory, adapts to the practical realities of Chinese stomatological outpatient practice, and addresses existing monitoring blind spots holds substantial theoretical and practical significance for advancing outpatient care quality and patient safety.

## 2 Materials and methods

### 2.1 Sources of information

**Figure 1:**
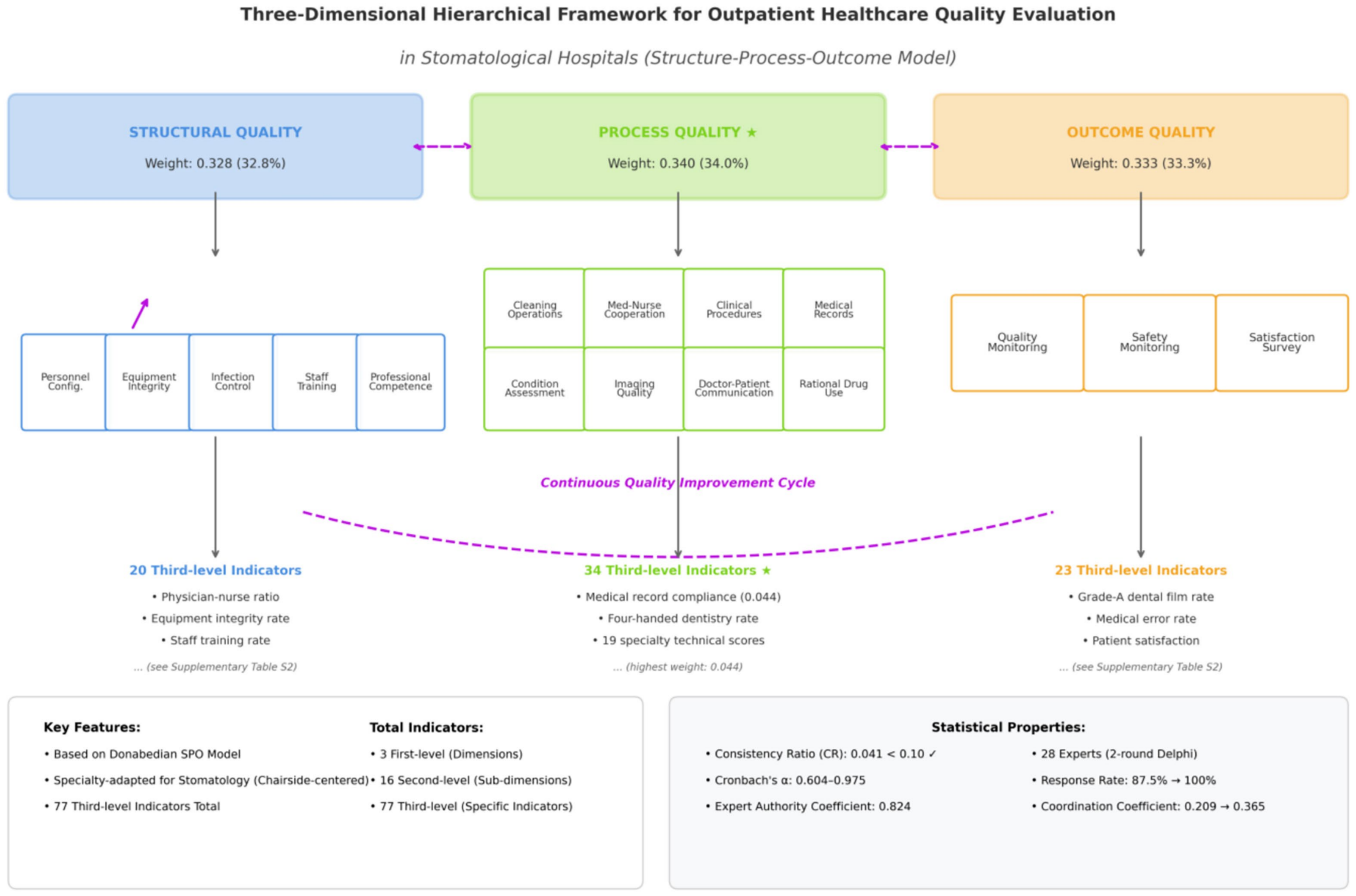
Three-dimensional hierarchical framework for outpatient healthcare quality evaluation in stomatological hospitals. The framework integrates Donabedian’s structure-process-outcome (SPO) model with stomatological specialty characteristics, comprising 3 first-level dimensions (with AHP-derived weights), 16 s-level sub-dimensions, and 77 third-level indicators. Bidirectional arrows indicate dynamic interactions and continuous quality improvement cycles.

A systematic literature search was conducted across CNKI, NCBI, and PubMed databases using the following keywords: “medical quality evaluation” “outpatient medical quality” “outpatient medical quality evaluation” “oral medical quality evaluation” “evaluation of oral medical quality” and “evaluation of oral clinical quality”. This initial search yielded 93 articles, of which 55 met inclusion criteria after screening for relevance. The selected literature encompassed medical quality evaluation methodologies, quality evaluation index systems for general hospitals, and current status of oral medical quality. Drawing upon established frameworks for general hospital quality evaluation and empirical findings from oral healthcare quality research, combined with standardized stomatology textbooks, we identified three quality dimensions—structural quality, process quality, and outcome quality—comprising 3 first-level indicators, 16 s-level indicators, and 77 third-level indicators (see in Figure 1).

### 2.2 Focus group discussion method

The specialized task force consisted of eight core members representing the administrative, management, dental practice, and nursing departments of a specialist dental hospital. Through multi-perspective brainstorming sessions, the group engaged in in-depth discussions on evaluating outpatient medical quality, covering the conceptual framework, research methodology, key indicators, scale dimensions, and terminology. The task force systematically reviewed the current state of research, reached consensus, and continued to optimize and refine the indicator system.

### 2.3 Delphi method

#### 2.3.1 Expert selection criteria

A purposive sampling approach combined with snowball sampling was employed to recruit expert panelists. Inclusion criteria comprised: (1) professional tenure: ≥10 years in clinical stomatology, nursing, or hospital administration, or ≥5 years in dental technology or pharmacy; (2) professional qualifications: associate senior professional title or above, or deputy section-level position or higher in functional/clinical departments; (3) geographic representation: experts were recruited from two tertiary grade-A stomatological hospitals in South China (Guangzhou Medical University Affiliated Stomatological Hospital) and Southwest China (Chongqing Medical University Affiliated Stomatological Hospital), balancing research accessibility with regional representation; (4) disciplinary distribution: ensuring coverage of four domains—clinical stomatology (target proportion ≥60%), nursing management (≥10%), hospital administration (≥15%), and medical technology/pharmacy (≤15%); and (5) exclusion criteria: individuals with publications expressing direct academic conflicts with this study’s objectives within the past 3 years, or those lacking Delphi participation experience and unwilling to undergo methodological training.

#### 2.3.2 Sample size justification

The final panel comprised 28 experts. Sample size determination was based on three considerations. First, Delphi methodology research indicates that when expert homogeneity is high, a sample of 20–30 participants satisfies statistical requirements with reliability comparable to samples of several hundred (14). Second, the recruited panel demonstrated high homogeneity (all from tertiary grade-A hospitals; 75% holding associate senior titles or above), rendering 28 participants sufficient to ensure precision in coordination coefficient estimation at 95% confidence level. Third, pilot testing confirmed this sample size maintained expert engagement (response rates: 87.5 and 100% across two rounds) while ensuring statistical stability.

#### 2.3.3 Indicator screening criteria

Indicator screening employed threshold analysis integrated with expert qualitative feedback. Quantitative criteria included: (1) importance score: indicators with arithmetic mean <4.0 on a 5-point Likert scale were eliminated; (2) coefficient of variation (CV): indicators with CV > 0.25 (indicating substantial disagreement) were flagged for discussion; and (3) full-score frequency: indicators with <50% of experts scoring 5 and mean <4.2 were considered for removal. Qualitative criteria encompassed: (1) textual expert feedback: indicators receiving explicit deletion or modification suggestions from ≥3 experts; (2) semantic ambiguity: indicators deemed ambiguous or difficult to quantify by the focus group; and (3) content redundancy: indicators with correlation coefficients >0.8 with existing items were merged.

Round-specific procedures: Round 1 applied both quantitative and qualitative criteria simultaneously, eliminating non-compliant indicators, merging similar items, and incorporating new indicators based on expert suggestions. Round 2 focused exclusively on indicators retained from Round 1, with particular scrutiny of indicators with CV > 0.20 that survived Round 1, requiring experts to reassess and justify retention. Only indicators simultaneously meeting mean ≥4.0 and CV ≤ 0.25 proceeded to weight calculation.

### 2.4 Analytic hierarchy process (AHP)

The Analytic Hierarchy Process (AHP) was employed to calculate indicator weights at all levels. Pairwise comparison matrices were constructed based on arithmetic mean importance scores from Round 2 Delphi consultation. The conversion rules for scale values were as follows: mean difference <0.25 = 1 (equally important); 0.25–0.75 = 2 (slightly more important); 0.75–1.25 = 3 (moderately more important); 1.25–1.75 = 4 (strongly more important); 1.75–2.25 = 5 (extremely more important); and >2.25 = 6–9 (progressively more important).

For first-level indicators, mean scores were: process quality 4.52, outcome quality 4.38, and structural quality 4.35. The difference between process and outcome quality was 0.14 (<0.25), warranting a scale value of 1; however, considering expert qualitative consensus favoring process quality and focus group deliberation, the scale value was adjusted to 2 (slightly more important). The resulting comparison matrix yielded weights of 0.328 for structural quality, 0.340 for process quality, and 0.333 for outcome quality, with a consistency ratio (CR) of 0.041 (<0.10), satisfying the consistency requirement. (Complete pairwise comparison matrices are provided in Supplementary material S1).

### 2.5 Statistical methods

Weights were calculated using the Sum-Product Method to approximate the maximum eigenvalue (λmax) and its corresponding normalized eigenvector. The procedure comprised three steps: (1) column normalization of the pairwise comparison matrix; (2) calculation of row averages to derive the weight vector; and (3) computation of λmax using the formula λmax = *Σ*[(AW)i/nwi], where (AW)i represents the ith element of the product matrix between the comparison matrix and weight vector. Consistency was assessed using the consistency index (CI) = (λmax−n)/(n − 1) and consistency ratio (CR) = CI/RI, with CR < 0.10 indicating satisfactory consistency.

Computational tools included Yaahp software (Version 12.5) for matrix operations and consistency testing, with cross-validation performed using R software (Version 4.2.1, Ahp package Version 0.3.1) to ensure result accuracy. Microsoft Excel 2016 was utilized for data organization and preliminary calculation verification.

## 3 Results

### 3.1 Expert engagement level

In the first round of this study, 32 questionnaires were distributed, and 28 valid questionnaires were collected, resulting in an 87.50% response rate. Following the design principles of the Delphi method, experts who expressed willingness to continue were invited to participate in the second round. All 28 experts agreed to participate, and 28 questionnaires were distributed and returned, yielding a response rate of 100%.

### 3.2 Basic information of experts

This study conducted two rounds of inquiries, inviting 28 experts with associate senior and senior titles from the Affiliated Stomatological Hospital of Guangzhou Medical University and the Affiliated Stomatological Hospital of Chongqing Medical University. The basic information of these experts is presented in Table 1.

**Table 1:** Basic information of experts.

**Parameter**	**Group**	***n***	**Percentage (%)**
Position	Deputy Head of Functional Department	4	14.3
	Head of Functional Department	5	17.9
	Deputy Head of Clinical/Medical Technology Department	10	35.7
	Head of Clinical/Medical Technology Department	9	32.1
Title	Intermediate professional title	7	25
	Associate senior professional title	11	39.3
	Senior professional title	10	35.7
Professional	Oral clinical care	3	10.7
	Oral clinical medicine	18	64.3
	Oral healthcare management	4	14.3
	Pharmacy	1	3.6
	Medical technology	1	3.6
	Others	1	3.6
Educational background	Undergraduate	8	28.6
	Master’s degree	9	32.1
	Doctoral degree	11	39.3

### 3.3 Expert authority level

The expert authority coefficient (Cr) is determined based on the expert’s judgment criteria (Ca) and familiarity level (Cs), using the formula Cr = (Ca + Cs) / 2. In this study, the judgment criteria score (Ca) was 0.943. For familiarity level, “very familiar,” “familiar,” “somewhat familiar,” “not very familiar,” and “very unfamiliar” were assigned values of 0.9, 0.7, 0.5, 0.3, and 0.1, with frequencies of 6, 17, 4, 2, and 0, respectively. The calculated expert familiarity (Cs) was 0.704, resulting in an expert authority coefficient of 0.824. As this value exceeds 0.7, it indicates high expert authority and reliable results. Details are provided in Table 2.

**Table 2:** Basis for expert judgement.

Criteria for judgement	Large		Medium		Small	
	Assignment	Frequency	Assignment	Frequency	Assignment	Frequency
Theoretical analysis	0.3	24	0.2	4	0.1	0
Practical experience	0.5	25	0.4	3	0.3	0
Peer understanding	0.1	20	0.1	5	0.1	3
Expert intuition	0.1	6	0.1	10	0.1	12

Kendall’s coefficient of concordance (W) was used to assess the consistency of expert evaluations. The results indicated that the W values for both rounds of consultation ranged from 0.209 to 0.365, all above 0.2, with *p-*values less than 0.05. This demonstrates that the experts’ correlation coefficients in both rounds were significant, indicating a good level of coordination among experts. Details are provided in Table 3.

**Table 3:** Coordination coefficients of inquired experts.

Parameters	1st round	2nd round
*W*	0.209	0.365
*X^2^*	743.561	427.022
*P*	0.000	0.000

### 3.4 Expert opinion concentration

After two rounds of Delphi expert consultations, the arithmetic mean values of all indicators ranged from 3.822 to 5. Only four indicators (4.17%) had mean values below 4 and a coefficient of variation less than 0.3, indicating a high degree of consensus among experts regarding the evaluation indicators for outpatient medical quality at specialized dental hospitals. Ultimately, three primary indicators, 16 secondary indicators, and 77 tertiary indicators were established.

### 3.5 Indicator screening results and weights

**Figure 2:**
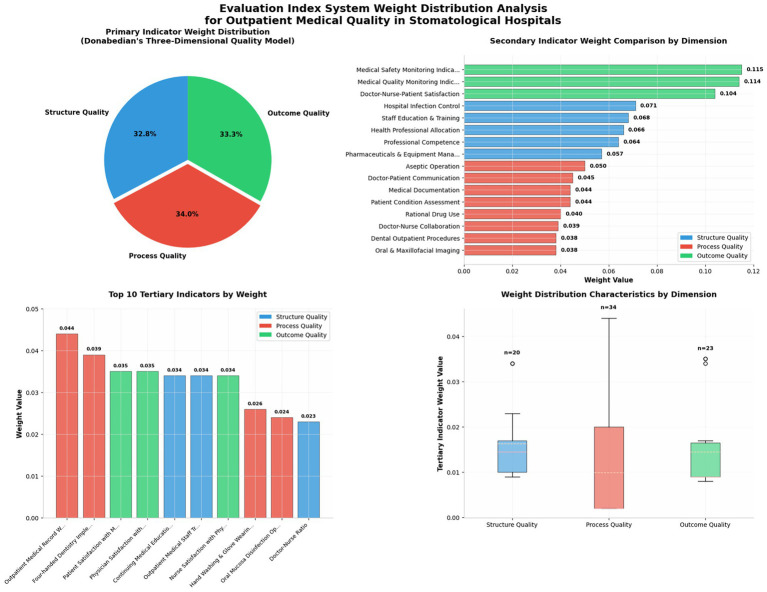
Evaluation index system weight distribution analysis for outpatient medical quality in stomatological hospitals.

This study employed the Analytic Hierarchy Process and Delphi method to construct an evaluation index system for stomatological hospital outpatient quality, with weights calculated using SPSS 24.0. For first-level indicators, structural quality, process quality, and outcome quality received weights of 0.328, 0.340, and 0.333, respectively (CI = 0.0213, RI = 0.520, CR = 0.0410 < 0.10), indicating satisfactory consistency. Process quality achieved the highest weight (0.340), though the marginal difference (<4%) across all three dimensions suggests balanced importance. This weight distribution aligns with three key considerations. First, it reflects the “process-oriented” nature of stomatological outpatient care. Characterized by “large outpatient volume, small inpatient capacity, and chairside-centered operations,” stomatological hospitals feature brief patient encounters where single-visit quality depends critically on procedural standardization. Unlike inpatient services with extended observation windows, outpatient outcomes are directly determined by process quality, justifying its prioritized weighting. Second, it embodies a “prevention-first” quality management philosophy. Traditional monitoring over-relies on outcome indicators (e.g., complication rates) representing “post-hoc remediation.” This study shifts weight forward to emphasize preventing adverse events through standardized processes (medical record documentation, disinfection protocols, physician-patient communication), aligning with modern “zero harm” objectives. Third, it represents scientific equilibrium within the three-dimensional framework. The minimal weight variation (0.340 vs. 0.328) indicates expert consensus that optimal quality requires synergistic optimization across structure, process, and outcome—no single dimension alone ensures overall quality. This “emphasis within equilibrium” captures the essence of Donabedian’s three-dimensional quality model (see in Figure 2 and Tables 4–7).

**Table 4:** Comparison of characteristics across three quality dimensions (structure-process-outcome).

Metric	Structure	Process	Outcome
Primary weight	0.328 (32.8%)	0.340 (34.0%) *	0.333 (33.3%)
Indicator count	20	34 (Most)	23
Mean weight	0.0164	0.0100	0.0145
Weight range	0.009–0.034	0.002–0.044 *	0.008–0.035
Top indicator	Staff training (0.034)	Medical records (0.044) *	Patient satisfaction (0.035)

*indicates the highest weight.

**Table 5:** Structure quality indicators and weights primary indicator weight: 0.328 (32.8%).

Secondary indicator	Secondary weight	Tertiary indicator	Tertiary weight
Hospital infection control	0.071	Healthcare worker hand hygiene compliance rate	0.015
		Medical device disinfection and sterilization pass rate	0.014
		Building layout and disinfection facilities compliance rate	0.014
		Air culture pass rate	0.014
		Medical water inspection pass rate	0.014
Staff education and training	0.068	Outpatient medical staff training implementation rate	0.034
		Continuing medical education implementation rate	0.034
Health professional allocation	0.066	Doctor-nurse ratio	0.023
		Proportion of senior professional title physicians	0.023
		Proportion of physicians with master’s degree or above	0.020
Professional competence	0.064	Professional competence	0.016
		Professional ethics	0.016
		Psychological quality	0.016
		Work attitude	0.016
Pharmaceuticals and equipment management	0.057	Emergency and life support equipment integrity rate	0.010
		Medical equipment maintenance inspection pass rate	0.010
		Emergency drug integrity rate	0.010
		Proportion of commonly used drug procurement varieties	0.009
		Medical equipment integrity rate	0.009
		Outpatient department satisfaction with medical equipment	0.009

**Table 6:** Process quality indicators and weights primary indicator weight: 0.340 (34.0%) * HIGHEST.

Secondary indicator	Secondary weight	Tertiary indicator	Tertiary weight
Medical documentation	0.044	Outpatient medical record writing pass rate	0.044 *
Aseptic operation	0.050	Hand washing and glove wearing operation score	0.026
		Oral mucosa disinfection operation score	0.024
Doctor–patient communication	0.045	Informed consent form signing pass rate	0.023
		Health education awareness rate	0.022
Patient condition assessment	0.044	Cardiac function indicators interpretation accuracy	0.022
		Blood glucose and metabolite test interpretation accuracy	0.022
Rational drug use	0.040	Antimicrobial drug use rate	0.020
		Outpatient prescription pass rate	0.020
Physician–nurse collaboration	0.039	Four-handed dentistry implementation rate	0.039
Dental outpatient procedures	0.038	Tooth mobility examination score	0.002
		Community periodontal index examination score	0.002
		Pulp temperature test score	0.002
		Periodontal probing examination score	0.002
		Occlusal relationship examination score	0.002
		Pulp opening operation score	0.002
		*In vitro* molar multi-surface cavity preparation score	0.002
		Intraoral suturing operation score	0.002
		Tooth extraction operation score	0.002
		Posterior superior alveolar nerve block anesthesia score	0.002
		Inferior alveolar nerve block anesthesia score	0.002
		Alveolar abscess incision and drainage score	0.002
		Pit and fissure sealant operation score	0.002
		Maxillofacial bandage wrapping score	0.002
		Dental implant operation score	0.002
		Chronic periodontitis systemic treatment score	0.002
		All-ceramic crown restoration score	0.002
		Oral care operation score	0.002
		Personal safety protection score	0.002
Oral and maxillofacial imaging	0.038	Laboratory test result reporting standardization rate	0.008
		Oral CBCT accurate interpretation rate	0.008
		Grade A dental film rate	0.008
		Image retake rate	0.007
		Panoramic radiograph accurate interpretation rate	0.007

**Table 7:** Outcome quality indicators and weights primary indicator weight: 0.333 (33.3%).

Secondary indicator	Secondary weight	Tertiary indicator	Tertiary weight
Medical safety monitoring indicators	0.115	Foreign body ingestion into digestive tract rate	0.017
		Hospital-acquired infection occurrence rate	0.017
		Oral soft tissue injury rate	0.017
		Medical error and accident occurrence rate	0.016
		Common tooth extraction complication rate	0.016
		Root canal treatment instrument separation rate	0.016
		Medical (safety) adverse event reporting count	0.016
Medical quality monitoring indicators	0.114	Root canal treatment adequate filling rate	0.009
		Root canal retreatment rate	0.009
		Inlay rework rate	0.009
		Fixed prosthesis repair rate	0.009
		Fixed prosthesis rework rate	0.009
		Implant loss rate	0.009
		Implant crown loss rate	0.009
		Bracket bonding position accuracy rate	0.009
		Final orthodontic quality improvement index	0.009
		Periodontal treatment 6–8 weeks BOP positive site rate	0.009
		Orthodontic appliance repair rate	0.008
		1-year orthodontic quality improvement index	0.008
		Primary molar filling loss rate	0.008
Doctor–nurse–patient satisfaction	0.104	Patient satisfaction with medical staff and services	0.035
		Physician satisfaction with nurses	0.035
		Nurse satisfaction with physicians	0.034

For second-level indicators, all CR values are less than 0.1, indicating that the secondary indicator judgment matrix satisfies the consistency test. The calculated weights demonstrate consistency, with the medical safety monitoring indicator (0.115) and the medical quality monitoring indicator (0.114) having significantly higher weight coefficients than other secondary indicators. Together, they account for 68.8% of the final quality weight. This reflects the strategic positioning of dental specialty hospitals that equally emphasizes “bottom-line thinking” and “pursuit of excellence” in outpatient management: On one hand, dental procedures involve high-frequency invasive operations, small instruments prone to dislodgement, and elevated infection risks. The severe consequences and hidden nature of safety incidents—such as foreign body aspiration or healthcare-associated infections—mandate that safety monitoring must serve as an inviolable management baseline. On the other hand, the “millimeter-level precision” requirements of dental care (such as proper root canal filling, osseointegration of implants, and marginal adaptation of prosthetics) directly impact long-term treatment outcomes. Metrics like rework rates and success rates serve as dual safeguards for hospital efficiency and patient trust. Together, these form a dual-wheel “safety-quality” model that proactively aligns with DRG/DIP payment reform trends while reflecting the industry’s shift from “basic service accessibility” to “high-risk technical control and precision medical quality.” This provides a scientific evaluation framework for building a dental healthcare system characterized by “zero harm, zero defects, and high satisfaction”.

For third-level indicators, all CR values <0.10 confirmed satisfactory consistency. “Outpatient medical record documentation qualification rate” achieved the highest weight (0.044), indicating three critical functions: (1) Holographic quality carrier: Medical records in dental outpatient settings serve as comprehensive reflections of the entire care process beyond legal documentation, encapsulating patient assessment adequacy, diagnostic reasoning rigor, treatment plan standardization, informed consent compliance, and follow-up arrangement rationality within a single metric. (2) Risk management and legal protection: Dental procedures carry inherent risks (nerve injury, infection, instrument separation); standardized documentation provides primary evidence for medical dispute resolution. Experts assigned top weight recognizing that documentation quality directly correlates with legal risk exposure and patient safety incident traceability. (3) Quality control leverage point: Medical record review offers a cost-effective, scalable monitoring tool compared to direct clinical observation, encouraging hospitals to establish robust documentation audit systems using this “soft indicator” to drive improvements across clinical processes.

### 3.6 Verification of the reliability of the indicator system

Following the calculation, the Cronbach’s *α* coefficients for each dimension of the constructed indicator system ranged from 0.604 to 0.975. All values exceeded 0.6, indicating satisfactory reliability. As “Outpatient Medical Record Writing Compliance Rate” and “Four-Handed Procedure Implementation Rate” are the only tertiary indicators under their respective secondary indicators, internal consistency reliability could not be assessed for these individual indicators. Therefore, the internal consistency reliability of the corresponding secondary indicators was considered satisfactory (see Table 8).

**Table 8:** The Cronbach’s α coefficients for each dimension of the evaluation index system for outpatient healthcare quality in stomatological hospitals.

**Dimension**	**Number of items**	**Cronbach’s *α***
1 Element quality	5	0.693
1.1 Configuration of health professionals	3	0.750
1.2 Pharmaceutical instruments and equipment	6	0.670
1.3 Hospital infection control	5	0.673
1.4 Medical staff education and training	2	0.884
1.5 Professionalism	4	0.862
2 Process quality	8	0.720
2.1 Cleaning operation	2	0.846
2.2 Medical and nursing cooperation	1	/
2.3 Oral Clinic Treatment Procedures	19	0.975
2.4 Medical documentation	1	/
2.5 Outpatient condition assessment	2	0.841
2.6 Oral and maxillofacial imaging examination	5	0.624
2.7 Doctor-patient communication	2	0.604
2.8 Rational use of medicines	2	0.696
3 Final quality	3	0.645
3.1 Healthcare quality monitoring indicators	13	0.973
3.2 Medical safety monitoring indicators	7	0.632
3.3 Medical staff, patient satisfaction	3	0.708

## 4 Discussion

This study developed a three-dimensional evaluation index system for outpatient healthcare quality in stomatological hospitals based on the structure-process-outcome framework. Through literature review, expert consultation, and analytic hierarchy process analysis, a comprehensive indicator system consisting of three first-level indicators, 16 second-level indicators, and 77 third-level indicators was established. The results indicate that process quality received the highest weight among the three dimensions, followed by outcome quality and structural quality.

### 4.1 Comparison with international dental quality frameworks

The three-dimensional quality evaluation index system developed in this study demonstrates substantial divergence from mainstream international dental quality frameworks in both theoretical foundation and indicator design, reflecting distinctive localized characteristics and practice-oriented orientation.

Comparison with the ADA Dental Quality Alliance (DQA) System, the DQA system, established under the American Dental Association, relies primarily on administrative claims data, emphasizing accessibility indicators (e.g., annual dental examination rates among diabetic patients) and basic process measures within an insurance payment and accountability framework. By contrast, this study’s system offers three differentiated advantages: First, comprehensive quality dimensions. The DQA system lacks evaluation of structural quality elements such as institutional management systems and personnel emergency response capabilities (15). This study incorporates professional competence (technical competency, professional ethics, psychological quality, work attitude) and healthcare staff training into structural quality, emphasizing the foundational role of “human factors” in quality formation (16). Second, refined process quality. The DQA’s reliance on claims data precludes assessment of single-visit quality depth (e.g., medical record standardization, procedural compliance, communication adequacy) (17). This study implements direct process evaluation through 19 specialty-specific technical operation scores (encompassing endodontics, periodontics, prosthodontics, implantology, orthodontics) and four-handed dentistry execution rates. Third, enhanced specialty specificity. DQA indicators employ generic designs that fail to accommodate the “large outpatient volume, small inpatient capacity, chairside-centered operations” characteristics of stomatological practice (18). This study specifically incorporates specialty-specific indicators including grade-A dental film rates, image retake rates, and oral mucosal disinfection operation scores, ensuring greater clinical relevance.

Comparison with the WHO Oral Health Evaluation Framework: The WHO oral health evaluation framework emphasizes population-level epidemiological indicators (e.g., DMFT index, caries-free rate), applicable to public health surveillance rather than internal healthcare institution quality management. This study focuses on service delivery quality within healthcare institutions, incorporating disinfection and isolation compliance rates, hand hygiene compliance rates, and physician-nurse collaboration satisfaction into evaluation. This aligns with WHO’s advocacy that “service quality is the foundation of health outcomes” (19), while providing more direct management leverage. Notably, the WHO framework lacks monitoring of medical safety adverse events and medical record standardization (20). This study addresses these gaps through medical safety monitoring indicators (weight 0.115) and outpatient medical record documentation qualification rate (weight 0.044, highest among all third-level indicators).

Theoretical Alignment with Donabedian’s Three-Dimensional Quality Model: this study employs the “structure-process-outcome” three-dimensional quality model as its theoretical foundation, with deep customization for stomatological specialty characteristics. Compared with traditional SPO model applications in general hospitals (21), this study adds stomatology-specific equipment integrity rates (e.g., dental unit chairs, oral CBCT, implant motors) to structural quality; constructs a specialty-specific technical operation scoring system within process quality; and incorporates specialty indicators such as grade-A dental film rates into outcome quality. This adaptation translates generic quality theory into the stomatological outpatient context. Furthermore, the minimal weight variation across three dimensions (<4%; 0.340 vs. 0.328 vs. 0.333) reflects “emphasis within equilibrium”, consistent with Donabedian’s core principle of “synergistic optimization across structure, process, and outcome” (22).

### 4.2 Innovations and application value of the index system

#### 4.2.1 Theoretical innovations

This three-dimensional quality evaluation index system specifically addresses three core limitations in current stomatological outpatient quality monitoring in China, achieving three transformative shifts:

From “outcome evaluation” to “three-dimensional integration.” Traditional monitoring over-relies on outcome quality indicators. This study places structural, process, and outcome quality on equal footing, forming a closed-loop “structure-process-outcome” management system. Particularly through incorporating institutional responsibility systems into structural quality evaluation, and four-handed dentistry execution rates and specialty-specific technical operation scores into process quality evaluation, quality control checkpoints shift forward to daily clinical practice, achieving a fundamental transformation from “outcome tracing” to “process prevention.”

From “generic framework” to “specialty-customized” precision adaptation. Structural quality adds stomatology-specific equipment integrity rates; process quality constructs a 19-item specialty-specific technical operation scoring system, highlighting four-handed dentistry execution rates as a distinctive physician-nurse collaboration model in stomatological outpatient care; outcome quality incorporates specialty-specific indicators such as grade-A dental film rates and image retake rates. This “specialty-specific adaptation” translates generic quality theory into the stomatological outpatient context.

From “single-point monitoring” to “systematic integration.” Through Delphi and AHP methods, 77 third-level indicators are integrated into 16 s-level and 3 first-level indicators, forming a quantifiable, comparable, and trackable comprehensive system. Particularly through the weighting of outpatient medical record documentation qualification rate (0.044, highest among all third-level indicators) and medical safety monitoring indicators (0.115 at second level), the system emphasizes medical documentation as a core process quality lever while highlighting patient safety as the outcome quality baseline, achieving systematic integration of key quality elements.

#### 4.2.2 Application value

For hospital administrators: This study provides a decision-support tool directly applicable to clinical management. The 16 s-level and 77 third-level indicators cover the complete outpatient workflow—from personnel configuration, equipment management, and institutional development to technical operations, documentation, and patient safety—addressing administrators’ practical dilemma regarding “what to evaluate and how to evaluate” (23). The AHP-derived weights provide quantitative evidence for resource allocation and priority setting: the highest weight for process quality (0.340) signals that administrators should shift quality control forward to the care delivery process, preventing adverse events through standardized documentation, enhanced four-handed dentistry, and strict disinfection protocols rather than relying solely on post-hoc outcome indicators (24).

For accreditation and review: this index system provides scientific evidence for the grading evaluation of stomatological hospital, specialty certification, and medical insurance payment standard development. Structural indicators including physician-nurse ratios, postgraduate degree proportions among outpatient physicians, and senior title proportions, alongside safety indicators such as medical error/incident rates and nosocomial infection rates, align with core requirements of the Tertiary Stomatological Hospital Review Standards (2022 Edition) (25). The quantifiable nature of indicators (Cronbach’s *α* 0.604–0.975, satisfactory reliability) enables regional quality monitoring and hospital performance evaluation, promoting standardization and homogenization of stomatological services (26).

For policy development: this study provides a management tool for addressing between stomatological resource shortage and growing demand in China. With the current dentist-to-population ratio of 1:7,768—below WHO standards—improving service quality within limited resources represents a critical policy challenge. This system identifies resource of the contradiction weaknesses through structural quality evaluation, optimizes existing resource allocation efficiency through process quality evaluation, and validates policy effectiveness through outcome quality evaluation (16). We recommend that medical insurance departments incorporate process quality indicators (e.g., medical record documentation qualification rates, rational drug use rates) into DRG/DIP payment assessment, incentivizing institutions to enhance the quality of their services rather than merely expanding service volume (27).

### 4.3 Implementation challenges and response strategies

Data collection challenges: the 77 third-level indicators involve multi-departmental data from clinical, nursing, technical, and administrative sources. Some indicators (e.g., specialty-specific technical operation scores) require professional personnel for on-site observation or medical record review, incurring substantial data collection costs (28). Response strategies include: developing automated data collection modules based on hospital information systems for real-time capture of objective indicators (equipment integrity rates, prescription qualification rates); and employing sampling inspection rather than comprehensive census for indicators requiring manual evaluation (e.g., operation scores), recommending 10–20% of cases or operation sampling per quarter (29).

Dynamic weight adjustment requirements: This study’s weights reflect expert subjective judgment and current healthcare environment quality priorities. As stomatological technology advances (e.g., widespread use of digital diagnosis and treatment) and policy environments evolve (e.g., medical insurance payment reforms), indicator weights require periodic revision (30). We recommend expert consultation rounds every 3–5 years for weight updates, while encouraging hospitals to implement 10–15% flexible adjustments to second-level indicator weights based on developmental stage (e.g., new vs. established institutions) (28).

Cross-institutional comparability limitations: direct horizontal comparison may be biased due to variations in patient disease composition, equipment configuration, and personnel structure across regions and hospital levels. We recommend employing risk adjustment methods considering case mix index (CMI) and hospital level factors for hospital ranking or certification applications; or adopting benchmarking approaches comparing with the same level and in the same region institutions (31).

Professional training requirements: accurate application depends on evaluators’ professional competence, particularly for specialty-specific technical operation scores requiring relevant clinical background. We recommend tiered training: clinical department quality control officers focusing on medical record standards and operation scoring criteria; functional department managers emphasizing data collection methods and statistical analysis skills; and senior executives prioritizing strategic interpretation of indicator systems and resource optimization methods (32).

### 4.4 Study limitations

First, limited expert representativeness. All 28 experts were recruited from two institutions (Guangzhou Medical University Affiliated Stomatological Hospital and Chongqing Medical University Affiliated Stomatological Hospital), with concentrated geographic distribution and excessive senior title representation (75%), potentially inadequately reflecting frontline intermediate-title practitioners’ perspectives and practices from other regions (e.g., North and East China).

Second, insufficient empirical validation. The indicator system was constructed based on expert subjective judgment without large-scale clinical data validation. The measurability and sensitivity of certain indicators (e.g., four third-level indicators under “professional competence”) require further testing.

Third, absence of patient perspectives. Delphi consultation included only healthcare professionals and administrators, without patient representatives, potentially inadequately addressing patient experience and needs.

### 4.5 Future validation plans

External validation: Incorporate experts from geographically diverse premier stomatological institutions (Peking University School of Stomatology, West China School of Stomatology Sichuan University) for Round 3 Delphi consultation or focus group interviews to validate cross-regional applicability.

Empirical testing: Select 3–5 hospitals across different levels (tertiary grade-A, tertiary grade-B, secondary) for 6–12 month pilot applications, testing indicator operability and sensitivity through actual data.

Stakeholder expansion: Include patient representatives and health administrative personnel in indicator weight revision, reflecting multi-center governance perspectives.

## 5 Conclusion

This study successfully constructed a stomatological hospital outpatient medical quality evaluation index system comprising 3 first-level, 16 s-level, and 77 third-level indicators with determined weights, based on the “structure-process-outcome” three-dimensional quality theory through literature review, focus group discussion, two-round Delphi expert consultation, and AHP. The system demonstrates three core strengths: (1) scientific theoretical framework with deep integration of the three-dimensional quality model and stomatological specialty characteristics, overcoming limitations of existing monitoring systems in dimensional singularity and insufficient specialty specificity (33). (2) comprehensive indicator coverage achieving full outpatient workflow evaluation across structural, process, and outcome quality, remedying traditional systems’ outcome-heavy, process-light deficiencies (34). (3) rational weight distribution with slightly elevated process quality weight (0.340) reflecting the “process-oriented” nature of stomatological outpatient care, and highest weight for outpatient medical record documentation qualification rate (0.044) highlighting medical documentation’s legal risk management and quality leverage functions (35).

This index system provides stomatological hospitals with a directly applicable quality management tool for internal quality improvement, departmental performance assessment, accreditation preparation, and policy reference. Immediate next steps include: (1) pilot testing across 3–5 hospitals of different levels for 6–12 months to validate operability, sensitivity, and reliability/validity through actual data (36). (2) Tool development of information-based evaluation systems for automated data collection, analysis, and feedback (37). (3) Dynamic optimization with 3–5 year revision cycles based on pilot feedback and healthcare environment evolution (22), ensuring its sustained applicability. Future research should expand expert consultation scope, incorporate patient perspectives, and conduct predictive validity studies verifying associations with patient health outcomes (33).

## Data Availability

The original contributions presented in the study are included in the article/Supplementary material, further inquiries can be directed to the corresponding author/s.
