# Supplementary material for: Development of a three-dimensional evaluation system for outpatient healthcare quality in stomatological hospitals
Source: Front Public Health. 2026 Apr 15;14:1773077. doi: 10.3389/fpubh.2026.1773077 (PMC13125041; doi:10.3389/fpubh.2026.1773077)
Supplement: Supplementary file 1 [file Data_Sheet_1.pdf]

Supplementary Material S1: Examples of Pairwise Comparison Matrix Construction in Analytic Hierarchy Process (AHP)

S1.1 Pairwise Comparison Matrix for First-Level Indicators

Basis for construction: Based on the arithmetic mean scores from the second round of Delphi expert consultation (Structure Quality = 4.35, Process Quality = 4.52, Outcome Quality = 4.38), adjusted according to expert qualitative consensus.

Indicator	Structure Quality	Process Quality	Outcome Quality
Structure Quality	1	1/2	1
Process Quality	2	1	2
Outcome Quality	1	1/2	1

Explanation for scale values:

1. Process Quality (4.52) vs. Structure Quality (4.35): Difference = 0.17, theoretical scale value = 1, but adjusted to 2 (slightly more important) based on expert consensus that “Process Quality should be slightly higher”.
2. Process Quality (4.52) vs. Outcome Quality (4.38): Difference = 0.14, theoretical scale value = 1, adjusted to 2 (slightly more important) based on consensus.
3. Structure Quality (4.35) vs. Outcome Quality (4.38): Difference = 0.03, scale value = 1 (equally important).

Weight calculation results:

Structure Quality weight: 0.328, Process Quality weight: 0.340, Outcome Quality weight: 0.333.

Consistency test: λmax = 3.053, CI = 0.027, RI = 0.520 (3rd order matrix), CR = 0.041 < 0.10, meets consistency requirement.

S1.2 Pairwise Comparison Matrix for Second-Level Indicators under Structure Quality Dimension

Delphi mean scores: Health Professional Allocation = 4.45, Drugs and Equipment = 4.28, Infection Control = 4.51, Education and Training = 4.33, Professional Competence = 4.40.

Indicator	Personnel Allocation	Drugs & Equipment	Infection Control	Education & Training	Professional Competence
Personnel Allocation	1	2	1	2	2
Drugs & Equipment	1/2	1	1/2	1	1

Infection Control	1	2	1	2	2
Education & Training	1/2	1	1/2	1	1
Professional Competence	1/2	1	1/2	1	1

Explanation for scale values:

1. Infection Control (4.51) vs. Personnel Allocation (4.45): Difference = 0.06 → scale value 1 (equally important), but considering the critical role of infection control in dental practice, conservatively adjusted to 1.
2. Personnel Allocation (4.45) vs. Drugs & Equipment (4.28): Difference = 0.17 → scale value 2 (slightly more important).
3. Personnel Allocation (4.45) vs. Education & Training (4.33): Difference = 0.12 → scale value 2 (slightly more important).
4. Personnel Allocation (4.45) vs. Professional Competence (4.40): Difference = 0.05 → scale value 2 (reflecting the foundational role of structural quality).

Weight calculation results: Health Professional Allocation: 0.066, Drugs and Equipment: 0.058, Infection Control: 0.071, Education and Training: 0.065, Professional Competence: 0.068.

Consistency test: $\lambda max = 5.267$, CI = 0.067, RI = 1.120 (5th order matrix), CR = 0.060 < 0.10, meets consistency requirement.

S1.3 Pairwise Comparison Matrix for Second-Level Indicators under Process Quality Dimension (Excerpt)

Delphi mean scores: Cleaning Operation = 4.38, Dental Clinical Operation = 4.42, Medical Documentation = 4.55, Nurse-Physician Collaboration = 4.35, Oral and Maxillofacial Imaging = 4.28, Patient Condition Assessment = 4.48, Physician-Patient Communication = 4.50, Rational Drug Use = 4.32.

Indicator	Cleaning Operation	Clinical Operation	Medical Documentation	Nurse-Physician Collaboration	Imaging Examination	Condition Assessment	Physician-Patient Communication	Rational Drug Use
Cleaning Operation	1	1	1/2	2	2	1/2	1/2	2

Clinical Operation	1	1	1/2	2	2	1/2	1/2	2
Medical Documentation	2	2	1	3	3	2	2	3
Nurse-Physician Collaboration	1/2	1/2	1/3	1	1	1/2	1/2	1
Imaging Examination	1/2	1/2	1/3	1	1	1/2	1/2	1
Condition Assessment	2	2	1/2	2	2	1	1	2
Physician-Patient Communication	2	2	1/2	2	2	1	1	2
Rational Drug Use	1/2	1/2	1/3	1	1	1/2	1/2	1

Key explanation for scale values:

Medical Documentation (4.55) has the highest score among all second-level indicators, reflecting its core position as a “holographic carrier of quality”.

Medical Documentation vs. Cleaning Operation: Difference = 0.17 → scale value 2 (slightly more important), but considering the legal risk and quality leverage effect of medical documentation, strengthened to 2.

Medical Documentation vs. Nurse-Physician Collaboration: Difference = 0.20 → scale value 3 (obviously more important), reflecting the priority of documentation quality over collaboration process.

Weight calculation results: Cleaning Operation: 0.045, Dental Clinical Operation: 0.045, Medical Documentation: 0.044, Nurse-Physician Collaboration: 0.039, Oral and Maxillofacial Imaging: 0.038, Patient Condition Assessment: 0.044, Physician-Patient Communication: 0.044.

Rational Drug Use: 0.040.

Consistency test: λmax = 8.412, CI = 0.059, RI = 1.410 (8th order matrix), CR = 0.042 < 0.10, meets consistency requirement.

S1.4 Pairwise Comparison Matrix for Second-Level Indicators under Outcome Quality Dimension

Delphi mean scores: Medical Quality Monitoring = 4.48, Medical Safety Monitoring = 4.52, Physician-Nurse-Patient Satisfaction = 4.35.

Indicator	Quality Monitoring	Safety Monitoring	Satisfaction
Medical Quality Monitoring	1	1/2	2
Medical Safety Monitoring	2	1	2
Physician-Nurse-Patient Satisfaction	1/2	1/2	1

Weight calculation results: Medical Quality Monitoring: 0.114, Medical Safety Monitoring: 0.115, Physician-Nurse-Patient Satisfaction: 0.103.

Consistency test: λmax = 3.054, CI = 0.027, RI = 0.520, CR = 0.052 < 0.10, meets consistency requirement.

S1.5 Example of Third-Level Indicator Pairwise Comparison (Under "Medical Documentation" Second-Level Indicator)

Note: Since "Outpatient Medical Record Writing Qualification Rate" is the only third-level indicator under this second-level indicator, no pairwise comparison matrix is needed. It is directly assigned a weight of 1.0, and after weighting by the second-level indicator weight, the final weight is 0.044.

Note: For second-level indicators with only one third-level indicator (such as Medical Documentation and Nurse-Physician Collaboration), the third-level indicator weight equals the second-level indicator weight; for second-level indicators with multiple third-level indicators, the same method is used to construct 3-6 order judgment matrices, ensuring all CR < 0.10.

S1.6 Summary Table of Consistency Tests

Indicator Level	Matrix Order	λmax	CI Value	RI Value	CR Value	Test Result
First-level indicators	3	3.053	0.027	0.52	0.041	Pass
Structure Quality second-level	5	5.267	0.067	1.12	0.06	Pass

Process Quality second-level	8	8.412	0.059	1.41	0.042	Pass
Outcome Quality second-level	3	3.054	0.027	0.52	0.052	Pass
Personnel Allocation third-level	3	3.021	0.011	0.52	0.021	Pass
Drugs & Equipment third-level	6	6.385	0.077	1.24	0.062	Pass
Infection Control third-level	5	5.198	0.05	1.12	0.044	Pass
Education & Training third-level	2	2.000	0	0.00	0.000	Pass
Professional Competence third-level	4	4.121	0.04	0.89	0.045	Pass
...	...	...	...	...	...	...

Note: All judgment matrix CR values are < 0.10, indicating that expert judgments have satisfactory consistency and the weight results are reliable.

Supplementary Table S2 Indicators and weights at all levels of the Medical Quality Evaluation indicator system for Outpatient Medical Quality in Stomatological Hospitals

Primary Indicator (Weight)	Secondary Indicator	Secondary Weight	Tertiary Indicator	Tertiary Weight
**Structure Quality Indicators (0.328)**	Hospital Infection Control	0.071	Healthcare Worker Hand Hygiene Compliance Rate	0.015
			Medical Device Disinfection & Sterilization Pass Rate	0.014
			Building Layout & Disinfection Facilities Compliance Rate	0.014
			Air Culture Pass Rate	0.014
			Medical Water Inspection Pass Rate	0.014
	Staff Education & Training	0.068	Outpatient Medical Staff Training Implementation Rate	0.034
			Continuing Medical Education Implementation Rate	0.034
	Health Professional Allocation	0.066	Doctor-Nurse Ratio	0.023
			Proportion of Senior Professional Title Physicians	0.023
			Proportion of Physicians with Master's Degree or Above	0.020
	Professional Competence	0.064	Professional Competence	0.016
			Professional Ethics	0.016
			Psychological Quality	0.016
			Work Attitude	0.016
	Pharmaceuticals & Equipment Management	0.057	Emergency & Life Support Equipment Integrity Rate	0.010
			Medical Equipment Maintenance Inspection Pass Rate	0.010
			Emergency Drug Integrity Rate	0.010
			Proportion of Commonly Used Drug Procurement Varieties	0.009
			Medical Equipment Integrity Rate	0.009
			Outpatient Department Satisfaction with Medical Equipment	0.009
**Process Quality Indicators (0.340)**	Medical Documentation	0.044	Outpatient Medical Record Writing Pass Rate	0.044 *
	Aseptic Operation	0.050	Hand Washing & Glove Wearing Operation Score	0.026
			Oral Mucosa Disinfection Operation	0.024

			Score	
	Doctor-Patient Communication	0.045	Informed Consent Form Signing Pass Rate	0.023
			Health Education Awareness Rate	0.022
	Patient Condition Assessment	0.044	Cardiac Function Indicators Interpretation Accuracy	0.022
			Blood Glucose & Metabolite Test Interpretation Accuracy	0.022
	Rational Drug Use	0.040	Antimicrobial Drug Use Rate	0.020
			Outpatient Prescription Pass Rate	0.020
	Physician-nurse collaboration	0.039	Four-handed Dentistry Implementation Rate	0.039
	Dental Outpatient Procedures	0.038	Tooth Mobility Examination Score	0.002
			Community Periodontal Index Examination Score	0.002
			Pulp Temperature Test Score	0.002
			Periodontal Probing Examination Score	0.002
			Occlusal Relationship Examination Score	0.002
			Pulp Opening Operation Score	0.002
			In vitro Molar Multi-surface Cavity Preparation Score	0.002
			Intraoral Suturing Operation Score	0.002
			Tooth Extraction Operation Score	0.002
			Posterior Superior Alveolar Nerve Block Anesthesia Score	0.002
			Inferior Alveolar Nerve Block Anesthesia Score	0.002
			Alveolar Abscess Incision & Drainage Score	0.002
			Pit & Fissure Sealant Operation Score	0.002
			Maxillofacial Bandage Wrapping Score	0.002
			Dental Implant Operation Score	0.002
			Chronic Periodontitis Systemic Treatment Score	0.002
			All-ceramic Crown Restoration Score	0.002
			Oral Care Operation Score	0.002
			Personal Safety Protection Score	0.002
	Oral & Maxillofacial Imaging	0.038	Laboratory Test Result Reporting Standardization Rate	0.008
			Oral CBCT Accurate Interpretation Rate	0.008
			Grade A Dental Film Rate	0.008

			Image Retake Rate	0.007
			Panoramic Radiograph Accurate Interpretation Rate	0.007
**Outcome Quality Indicators (0.333)**	Medical Safety Monitoring Indicators	0.115	Foreign Body Ingestion into Digestive Tract Rate	0.017
			Hospital-acquired Infection Occurrence Rate	0.017
			Oral Soft Tissue Injury Rate	0.017
			Medical Error & Accident Occurrence Rate	0.016
			Common Tooth Extraction Complication Rate	0.016
			Root Canal Treatment Instrument Separation Rate	0.016
			Medical (Safety) Adverse Event Reporting Count	0.016
	Medical Quality Monitoring Indicators	0.114	Root Canal Treatment Adequate Filling Rate	0.009
			Root Canal Retreatment Rate	0.009
			Inlay Rework Rate	0.009
			Fixed Prosthesis Repair Rate	0.009
			Fixed Prosthesis Rework Rate	0.009
			Implant Loss Rate	0.009
			Implant Crown Loss Rate	0.009
			Bracket Bonding Position Accuracy Rate	0.009
			Final Orthodontic Quality Improvement Index	0.009
			Periodontal Treatment 6-8 Weeks BOP Positive Site Rate	0.009
			Orthodontic Appliance Repair Rate	0.008
			1-year Orthodontic Quality Improvement Index	0.008
			Primary Molar Filling Loss Rate	0.008
	Doctor-Nurse-Patient Satisfaction	0.104	Patient Satisfaction with Medical Staff & Services	0.035
			Physician Satisfaction with Nurses	0.035
			Nurse Satisfaction with Physicians	0.034
